# Translation and validation of the Chinese version of the maternal postpartum stress scale

**DOI:** 10.1186/s12884-023-05990-y

**Published:** 2023-09-22

**Authors:** Yanchi Wang, Qian Gao, Jin Liu, Feng Zhang, Xujuan Xu

**Affiliations:** 1 Affiliated Nantong Hospital of Shanghai University (The Sixth People’s Hospital of Nantong), Nantong, Jiangsu China; 2 Medical School of Nantong University, Nantong, Jiangsu China; 3 Department of Nursing, Affiliated Hospital of Nantong University, Nantong, 226001 Jiangsu China

**Keywords:** Maternal Postpartum stress scale, Reliability, Validity, Exploratory factor analysis, Confirmatory factor analysis

## Abstract

**Objective:**

To translate the Maternal Postpartum Stress Scale (MPSS) into Chinese and validate its psychometric properties in postpartum women.

**Methods:**

A total of 406 postpartum women were recruited from six hospitals in Nantong, Jiangsu Province, China. Cronbach’s α co-efficient, split-half reliability, and test-retest reliability were used to evaluate the reliability of the translated scale. Exploratory factor analysis (EFA) and confirmatory factor analysis (CFA) were used to evaluate the structural validity of the scale. The Edinburgh Postnatal Depression Scale, Depression Anxiety Stress Scale-21 anxiety dimension, and Perceived Stress Scale were used as calibration scales to measure the correlation of MPSS. All data were analyzed using SPSS 25.0 and Amos 24.0.

**Results:**

The Cronbach’s α co-efficient of the Chinese version of MPSS and its three dimensions were 0.940 and 0.882–0.911, respectively. The split-half reliability was 0.825, and the test-retest reliability was 0.912. The scale’s content validity index was 0.926. Three common factors were extracted from the EFA. The CFA validated the explored 3-factor structure, and the indicators were fitted well (χ2/Df = 2.167, comparative fit index = 0.918, Tucker–Lewis index = 0.907, incremental fit index = 0.919, and root mean square error of approximation = 0.075).

**Conclusion:**

The translated Chinese version of MPSS had suitable reliability and validity in assessing postpartum stress in Chinese women. The translated scale can also help with the early identification of postpartum stress and provide a scientific basis for the formulation of early personalized intervention measures. Overall, the scale has certain clinical value and practical significance for enhancing the physical and mental health of postpartum women. However, future studies including large, diverse populations are warranted.

**Supplementary Information:**

The online version contains supplementary material available at 10.1186/s12884-023-05990-y.

## Introduction

Postpartum is a critical period for the mother and child, which may negatively affect maternal health [1]. Postpartum stress is usually conceptualized as a negative psychological response to the numerous obligations associated with raising children, and its presence is the rule rather than the exception [2]. Women with postpartum stress have intense, excessive, and persistent concerns and fears regarding various situations such as transitioning into parenthood, the baby’s health, and their personal health [3, 4]. In addition, they tend to have self-doubt, thus challenging their sense of self-evaluation and eventually developing severe postpartum mental diseases, such as postpartum depression [5]. Postpartum stress is reportedly more common than postpartum depression and affects a considerable proportion of mothers [6]. Therefore, an in-depth study of the causes of postpartum stress and its interventions is crucial.

Postpartum stress is often unrecognized and underdiagnosed because it is frequently comorbid or co-occurs with numerous other psychiatric disorders, such as anxiety disorders and major depression [7, 8]. This makes the treatment even more challenging, resulting in low remission rates and worse prognosis. Although structured interviews are widely used to diagnose postpartum stress, they are often time-consuming and require trained professionals to administer them [9]. As the first step in screening and early detection of postpartum stress, self-report measures are good, efficient, and easier. Although Hung Postpartum Stress Scale (HPSS) [10] is reliable and valid in assessing postpartum stress, its application of some items is limited to the early postpartum period (within 6 weeks after delivery). In addition, there were relatively too much items in the scale. Therefore, developing effective, easy, reliable and specific assessment tools to timely and accurately assesses postpartum stress and effectively screen and control the symptoms of postpartum stress is crucial.

In 2021, Nakić Radoš et al. [11] developed the Maternal Postpartum Stress Scale (MPSS) to assess maternal stress during postpartum maternity leave. The introduction of MPSS has enriched the evaluation tools for postpartum stress and has helped understand maternal stress at various postpartum time points and identify women at high risk for postpartum stress, which may be beneficial for formulating personalized interventions. To the best of our knowledge, except for the original English version, the MPSS has not been validated in other ethnic groups. In this study, the English version of MPSS was translated into Chinese accompanied by cultural modification, and the psychometric properties of the translated version were validated in general Han Chinese postpartum women which were recruited from Nantong (Located in eastern China), Jiangsu Province, China.

## Methods

### Participants and procedures

Using the convenience sampling method, 406 postpartum women who underwent physical examination 42 days after delivery at six hospitals (The Affiliated Hospital of Nantong University, The First People’s Hospital of Nantong, The Second People’s Hospital of Nantong, The Third People’s Hospital of Nantong, The Affiliated Maternity and Child Health Care Hospital of Nantong University, and The Sixth People’s Hospital of Nantong) in Nantong, Jiangsu Province, China, between August and November 2022 were selected as research participants.

According to Kendall’s sample size calculation method, the sample size should be 5–10 times the number of items in the questionnaire [12]. Therefore, given that the MPSS comprises 22 items, the sample size required for our study was 110–220 participants. However, assuming a 20% dropout rate, the required sample size was 132–264 participants. Because the recruitment rate was higher than expected, we were able to obtain 425 participants for our study, which met the sample size requirements. Of 425, 406 participants completed the MPSS in Chinese version, with a response rate of 95.53%. The final participants were subsequently divided into two groups using the split-sampling random procedure: Group 1, which included 200 participants, was assigned to exploratory factor analysis (EFA), and Group 2, which included 206 participants, was assigned to confirmatory factor analysis (CFA) [13]. During the survey, standardized instructions were presented to the participants with detailed explanations of the content and purpose of the investigation. All participants were informed that the participation was voluntary, their responses were confidential, and they could withdraw at any time. The survey lasted for approximately 20–30 min.

The inclusion criteria were as follows: (a) maternal age ≥ 18 years; (b) no malformations or serious complications in the newborn; (c) willingness to cooperate with the survey, ability to communicate effectively, and ability to understand the contents of the questionnaire and complete it independently; and (d) voluntary participation. The exclusion criteria were as follows: (a) unmarried mothers; (b) preterm births or twins; (c) comorbid psychosomatic diseases, such as chronic urticaria, neurodermatitis, hyperthyroidism, migraine, muscle pain, rheumatoid arthritis, sleep disorders, and malignant tumors; (d) mental illnesses (other than depression and anxiety), severe organic diseases such as heart, liver, and kidney diseases, and severe complications during pregnancy; (e) low intelligence and inability to understand the contents of the questionnaire; and (f) poor compliance and unwilling to cooperate in the study.

Prior to the survey, written informed consent was also obtained from all participants. Ethical approval for this study was obtained from the Research Ethics Committee of the Affiliated Hospital of Nantong University (approval number: 2022-K50-01).

### Measures

A questionnaire consisting of sociodemographic questions and 22 items from the translated MPSS was constructed. The Edinburgh Postnatal Depression Scale (EPDS) [14] and Depression Anxiety Stress Scale-21 (DASS-21) [15] were applied to evaluate divergent validity, and the 10-item Perceived Stress Scale (PSS-10) [16] was applied to evaluate convergent validity.

#### MPSS

MPSS [11] is a reliable and valid instrument that measures self-reporting postpartum stress. It contains 22 items and three subscales: personal needs and fatigue (9 items), infant nurturing (7 items), and body changes and sexuality (6 items). Each item is scored on a 5-point Likert scale, ranging from 0 (not at all) to 4 (completely). A higher MPSS total score indicates a higher postpartum stress level.

#### Translation of the MPSS

After obtaining permission from the original authors, MPSS was translated into Chinese using the classical “backward and forward” procedure following a modified Brislin translation model [17]. Two independent bilingual Chinese native psychologists performed the forward translation of items from English into Chinese. Following discussion and revision by all the authors and translators, a consensus forward Chinese version of the instrument was developed. Subsequently, the reconciled forward version of the scale was back-translated into English by two independent bilingual psychologists who were blinded to the original English version. In the third step, the original and back-translated English versions of the scale were compared according to the cross-cultural adaptation guidelines of the scale [18] and reviewed by a 15-member expert panel, including sociologists, psychologists, nursing education experts, clinical experts, and obstetric nursing experts. The findings showed that the versions were semantically equivalent. Next, cognitive interviews were conducted with 30 postpartum women to investigate the comprehensibility of the Chinese version of MPSS, and revisions were made [19]. The English version of MPSS is presented in Supplementary Table 1

#### EPDS

The Chinese version of the EPDS [14] was used to assess postpartum depression status. The scale comprises 10 items [20]. Each item response is divided into four levels, reflecting the different severity of symptoms, from “never” to “always” with a score of 0–3, respectively. The scores of the 10 items were summed to obtain the total individual score, which ranged from 0 to 30. We used 10 as the cutoff point [21].

#### DASS-21

DASS-21 [15] was used to measure depressive, anxiety, and stress symptoms among participants. It comprises 21 self-reporting items, with 7 items each in the 3 subscales: depression, anxiety, and stress. Respondents rate each item ranging from 0 (did not apply to me at all) to 3 (applied to me very much) in the past week. To ensure compatibility with DASS-42, the DASS-21 score is multiplied by 2. Psychometric properties of the scale in assessing depression, anxiety, and stress were well established, including among pregnant and postpartum women. In this study, the focus is on the anxiety dimension of the scale [22].

#### PSS-10

The validated Chinese version of the PSS-10 [16], which has been widely used in clinical research to measure general stress levels, was used to assess stress severity. Each item is rated on a 5-point scale, ranging from 0 (never) to 4 (very often), with a total score ranging from 0 to 40, whereby a higher score indicates a higher stress level [23].

### Statistical analyses

The EFA and CFA were used to explore and verify the potential factor structure of the scale, respectively. The scale is suitable for EFA only when the Kaiser-Meyer-Olkin (KMO) > 0.6 and Bartlett’s sphericity test were statistically significant (*P* < 0.05) [24]. Amos software (24.0) was used to validate the consistency of the model structure with the explored factor structure. These indices were used to examine the model’s goodness of fit: comparative fit index (CFI). When Chi-square/Df (χ2/Df) was < 3.000, root mean square residual was < 0.050, root mean square error of approximation (RMSEA) was < 0.080, and value incremental fit index (IFI), Tucker–Lewis index (TLI), and CFI exceeded 0.900 [25], the model was well fitted. Cronbach’s α coefficient was used to determine the internal consistency reliability of the scale. Cronbach’s α coefficient exceeding 0.7 was considered acceptable [26]. The two-sided chi-squared test was used to statistically evaluate differences in demographic characteristics between the EFA and CFA groups. Student’s t-test was used to statistically evaluate differences in total MPSS and subscale scores for postpartum women in different statuses. All data were analyzed using SPSS 25.0 and Amos 24.0, with a significant α threshold of 0.05 (two-tailed).

## Results

### Demographic information

The characteristics of participants in the EFA (n = 200) and CFA (n = 206) groups are summarized in Table 1. All the variables were comparable between both groups (*P* > 0.05).

**Table 1:** Characteristics of the subjects enrolled in this study

Variables		EFA^1^	CFA^2^	*P*		Variables		EFA^1^	CFA^2^	*P*
		(n = 200)	(n = 206)					(n = 200)	(n = 206)	
Age	< 30	106(53.00%)	122(59.22%)	0.206		Years of marriage	≤ 5	160(80.00%)	164(79.61%)	0.922
	≥ 30	94(47.00%)	84(40.78%)				> 5	40(20.00%)	42(20.39%)	
Educational level	Below bachelor	75(37.50%)	83(40.29%)	0.564		Abortion history	No	136(68.00%)	138(66.99%)	0.828
(PE ^3^)	Bachelor or above	125(62.50%)	123(59.71%)				Yes	64(32.00%)	68(33.01%)	
Educational level	Below bachelor	91(45.50%)	80(38.83%)	0.174		Parity	Primipara	154(77.00%)	163(79.13%)	0.605
(husband)	Bachelor or above	109(54.50%)	126(61.17%)				Multipara	46(23.00%)	43(20.87%)	
Family monthly income (CNY)	< 5000	5(2.50%)	10(4.85%)	0.259		Assisted reproduction	No	173(86.50%)	172(83.50%)	0.397
	5000–10,000	77(38.50%)	64(31.07%)				Yes	27(13.50%)	34(16.50%)	
	10,001–20,000	84(42.00%)	99(48.06%)			BMI	Normal	123(61.50%)	122(59.23%)	0.861
	> 20,000	34(17.00%)	33(16.02%)				Overweight	46(23.00%)	52(25.24%)	
Medical	No	24(12.00%)	32(15.53%)	0.302			Obesity	31(15.50%)	32(15.53%)	
insurance	Yes	176(88.00%)	174(84.47%)			Complications of pregnancy	No	138(69.00%)	143(69.42%)	0.927
Mode of Feeding	Breast-feeding	80(40.00%)	83(40.29%)	0.829			Yes	62(31.00%)	63(30.58%)	
	Mixed-feeding	99(49.50%)	105(50.97%)			Birth weight	< 3 kg	37(18.50%)	32(15.53%)	0.323
	formula-feeding	21(10.50%)	18(8.74%)				3-4 kg	147(73.50%)	149(72.33%)	
Mode of delivery	Vaginal	83(41.50%)	96(46.60%)	0.462			> 4 kg	16(8.00%)	25(12.14%)	
	Vaginal (lateral episiotomy)	39(19.50%)	32(15.53%)			Infant caretaker	Only PE ^3^	56(28.00%)	54(26.21%)	0.686
	Cesarean section	78(39.00%)	78(37.86%)				Involving others	144(72.00%)	152(73.79%)	

^1^ EFA: exploratory factor analysis;

^2^ CFA: confirmatory factor analysis;

^3^ PE: postpartum women

**Fig. 1:**
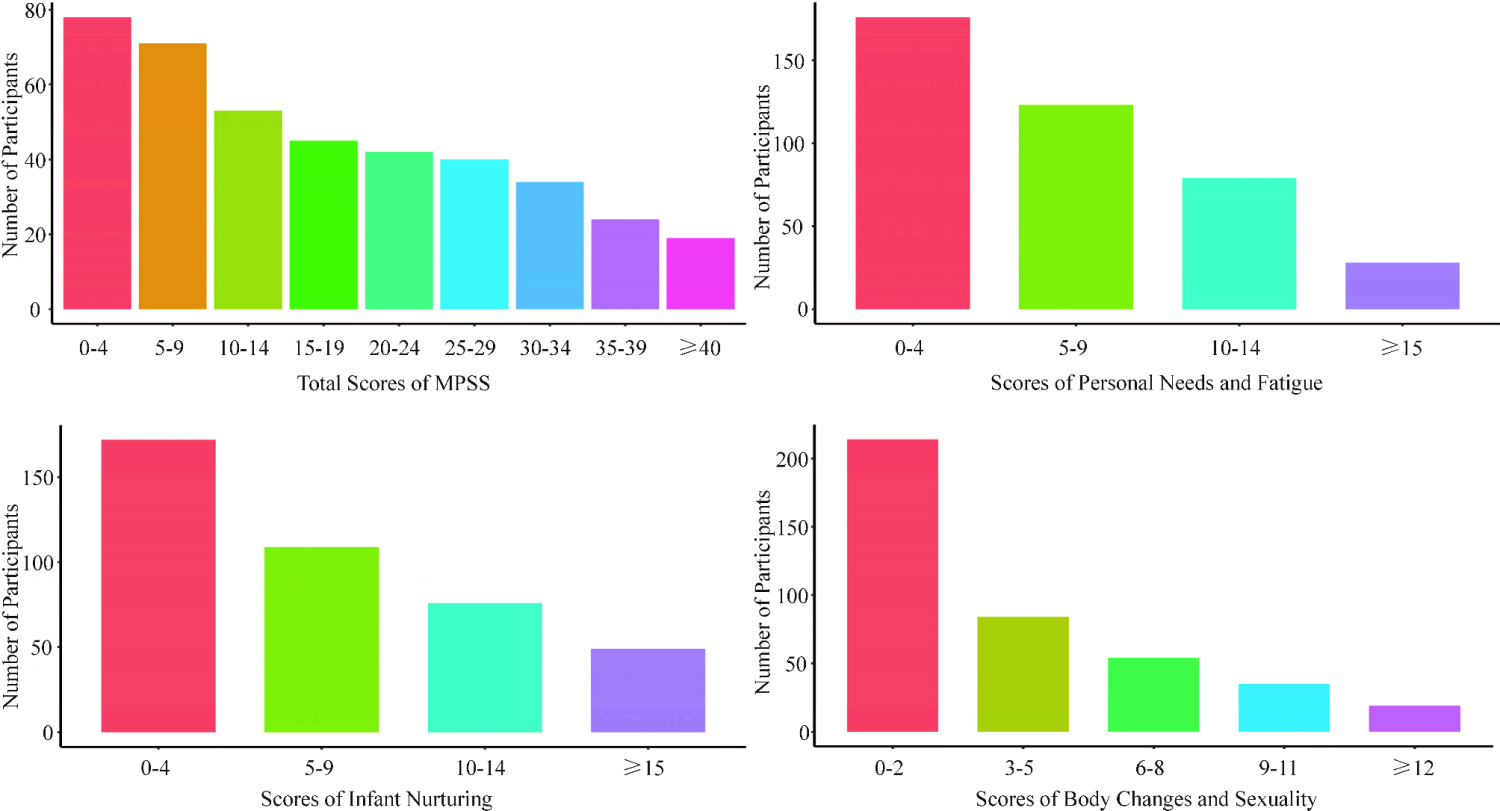
Number of participants in each MPSS and subscale groups

Among the total 406 participants, the median score of the MPSS was 15, while the median scores of the three subscales of the MPSS were 5 (personal needs and fatigue), 6 (infant nurturing), and 3 (body changes and sexuality), respectively. Details of the number of participants in each score group were showed in Fig. 1.

### Item analysis

The Chinese version of MPSS of the 406 survey respondents was sorted by their score, with those in the top 27% categorized into the high-stress group and those in the bottom 27% as the low-stress group. The mean of each item score in both groups was calculated. The two-tailed independent samples t-test showed a significant difference between the items in the two groups (*P* < 0.05). The specific statistical results are shown in Supplementary Table 2. The correlation analysis results (Table 2) revealed that Pearson’s correlation coefficient among all items in the scale was > 0.2. The results of the discrete trend method (Supplementary Table 3) showed that the coefficient of variation of each item was > 0.80, and all items met the inclusion criteria.

**Table 2:** The results of the discriminant validity analysis using the Pearson’ s correlation coefficient

	A1	A2	A3	A4	A5	A6	A7	A8	A9	A10	A11	A12	A13	A14	A15	A16	A17	A18	A19	A20	A21	A22	Total score
A1	1	0.623^**^	0.513^**^	0.650^**^	0.639^**^	0.586^**^	0.380^**^	0.469^**^	0.390^**^	0.353^**^	0.288^**^	0.255^**^	0.283^**^	0.371^**^	0.305^**^	0.271^**^	0.291^**^	0.381^**^	0.468^**^	0.412^**^	0.320^**^	0.399^**^	0.687^**^
A2	0.623^**^	1	0.613^**^	0.585^**^	0.587^**^	0.496^**^	0.445^**^	0.494^**^	0.485^**^	0.356^**^	0.271^**^	0.252^**^	0.326^**^	0.301^**^	0.270^**^	0.260^**^	0.262^**^	0.440^**^	0.501^**^	0.369^**^	0.407^**^	0.451^**^	0.679^**^
A3	0.513^**^	0.613^**^	1	0.577^**^	0.584^**^	0.490^**^	0.466^**^	0.446^**^	0.442^**^	0.352^**^	0.208^**^	0.341^**^	0.312^**^	0.351^**^	0.288^**^	0.246^**^	0.285^**^	0.345^**^	0.443^**^	0.377^**^	0.322^**^	0.355^**^	0.653^**^
A4	0.650^**^	0.585^**^	0.577^**^	1	0.839^**^	0.735^**^	0.456^**^	0.465^**^	0.391^**^	0.370^**^	0.275^**^	0.338^**^	0.362^**^	0.388^**^	0.349^**^	0.294^**^	0.312^**^	0.415^**^	0.479^**^	0.446^**^	0.337^**^	0.408^**^	0.743^**^
A5	0.639^**^	0.587^**^	0.584^**^	0.839^**^	1	0.759^**^	0.473^**^	0.466^**^	0.417^**^	0.390^**^	0.267^**^	0.338^**^	0.314^**^	0.362^**^	0.324^**^	0.296^**^	0.298^**^	0.417^**^	0.477^**^	0.420^**^	0.361^**^	0.411^**^	0.741^**^
A6	0.586^**^	0.496^**^	0.490^**^	0.735^**^	0.759^**^	1	0.548^**^	0.456^**^	0.426^**^	0.406^**^	0.295^**^	0.348^**^	0.334^**^	0.355^**^	0.319^**^	0.308^**^	0.334^**^	0.355^**^	0.483^**^	0.435^**^	0.412^**^	0.431^**^	0.726^**^
A7	0.380^**^	0.445^**^	0.466^**^	0.456^**^	0.473^**^	0.548^**^	1	0.465^**^	0.437^**^	0.385^**^	0.266^**^	0.230^**^	0.290^**^	0.299^**^	0.240^**^	0.247^**^	0.239^**^	0.337^**^	0.404^**^	0.328^**^	0.381^**^	0.343^**^	0.595^**^
A8	0.469^**^	0.494^**^	0.446^**^	0.465^**^	0.466^**^	0.456^**^	0.465^**^	1	0.699^**^	0.556^**^	0.507^**^	0.479^**^	0.357^**^	0.373^**^	0.353^**^	0.312^**^	0.320^**^	0.591^**^	0.746^**^	0.451^**^	0.594^**^	0.578^**^	0.752^**^
A9	0.390^**^	0.485^**^	0.442^**^	0.391^**^	0.417^**^	0.426^**^	0.437^**^	0.699^**^	1	0.532^**^	0.386^**^	0.441^**^	0.275^**^	0.334^**^	0.322^**^	0.274^**^	0.281^**^	0.527^**^	0.596^**^	0.367^**^	0.568^**^	0.524^**^	0.678^**^
A10	0.353^**^	0.356^**^	0.352^**^	0.370^**^	0.390^**^	0.406^**^	0.385^**^	0.556^**^	0.532^**^	1	0.367^**^	0.452^**^	0.350^**^	0.374^**^	0.362^**^	0.296^**^	0.266^**^	0.544^**^	0.549^**^	0.410^**^	0.554^**^	0.538^**^	0.653^**^
A11	0.288^**^	0.271^**^	0.208^**^	0.275^**^	0.267^**^	0.295^**^	0.266^**^	0.507^**^	0.386^**^	0.367^**^	1	0.489^**^	0.290^**^	0.309^**^	0.304^**^	0.348^**^	0.218^**^	0.415^**^	0.484^**^	0.297^**^	0.433^**^	0.427^**^	0.534^**^
A12	0.255^**^	0.252^**^	0.341^**^	0.338^**^	0.338^**^	0.348^**^	0.230^**^	0.479^**^	0.441^**^	0.452^**^	0.489^**^	1	0.320^**^	0.364^**^	0.349^**^	0.331^**^	0.241^**^	0.359^**^	0.512^**^	0.334^**^	0.413^**^	0.467^**^	0.572^**^
A13	0.283^**^	0.326^**^	0.312^**^	0.362^**^	0.314^**^	0.334^**^	0.290^**^	0.357^**^	0.275^**^	0.350^**^	0.290^**^	0.320^**^	1	0.611^**^	0.591^**^	0.528^**^	0.461^**^	0.452^**^	0.466^**^	0.509^**^	0.438^**^	0.400^**^	0.615^**^
A14	0.371^**^	0.301^**^	0.351^**^	0.388^**^	0.362^**^	0.355^**^	0.299^**^	0.373^**^	0.334^**^	0.374^**^	0.309^**^	0.364^**^	0.611^**^	1	0.844^**^	0.572^**^	0.526^**^	0.449^**^	0.431^**^	0.603^**^	0.406^**^	0.411^**^	0.672^**^
A15	0.305^**^	0.270^**^	0.288^**^	0.349^**^	0.324^**^	0.319^**^	0.240^**^	0.353^**^	0.322^**^	0.362^**^	0.304^**^	0.349^**^	0.591^**^	0.844^**^	1	0.604^**^	0.480^**^	0.466^**^	0.422^**^	0.526^**^	0.447^**^	0.425^**^	0.637^**^
A16	0.271^**^	0.260^**^	0.246^**^	0.294^**^	0.296^**^	0.308^**^	0.247^**^	0.312^**^	0.274^**^	0.296^**^	0.348^**^	0.331^**^	0.528^**^	0.572^**^	0.604^**^	1	0.374^**^	0.398^**^	0.383^**^	0.419^**^	0.434^**^	0.415^**^	0.565^**^
A17	0.291^**^	0.262^**^	0.285^**^	0.312^**^	0.298^**^	0.334^**^	0.239^**^	0.320^**^	0.281^**^	0.266^**^	0.218^**^	0.241^**^	0.461^**^	0.526^**^	0.480^**^	0.374^**^	1	0.455^**^	0.393^**^	0.698^**^	0.410^**^	0.367^**^	0.574^**^
A18	0.381^**^	0.440^**^	0.345^**^	0.415^**^	0.417^**^	0.355^**^	0.337^**^	0.591^**^	0.527^**^	0.544^**^	0.415^**^	0.359^**^	0.452^**^	0.449^**^	0.466^**^	0.398^**^	0.455^**^	1	0.671^**^	0.531^**^	0.633^**^	0.570^**^	0.718^**^
A19	0.468^**^	0.501^**^	0.443^**^	0.479^**^	0.477^**^	0.483^**^	0.404^**^	0.746^**^	0.596^**^	0.549^**^	0.484^**^	0.512^**^	0.466^**^	0.431^**^	0.422^**^	0.383^**^	0.393^**^	0.671^**^	1	0.519^**^	0.648^**^	0.592^**^	0.785^**^
A20	0.412^**^	0.369^**^	0.377^**^	0.446^**^	0.420^**^	0.435^**^	0.328^**^	0.451^**^	0.367^**^	0.410^**^	0.297^**^	0.334^**^	0.509^**^	0.603^**^	0.526^**^	0.419^**^	0.698^**^	0.531^**^	0.519^**^	1	0.468^**^	0.459^**^	0.704^**^
A21	0.320^**^	0.407^**^	0.322^**^	0.337^**^	0.361^**^	0.412^**^	0.381^**^	0.594^**^	0.568^**^	0.554^**^	0.433^**^	0.413^**^	0.438^**^	0.406^**^	0.447^**^	0.434^**^	0.410^**^	0.633^**^	0.648^**^	0.468^**^	1	0.674^**^	0.703^**^
A22	0.399^**^	0.451^**^	0.355^**^	0.408^**^	0.411^**^	0.431^**^	0.343^**^	0.578^**^	0.524^**^	0.538^**^	0.427^**^	0.467^**^	0.400^**^	0.411^**^	0.425^**^	0.415^**^	0.367^**^	0.570^**^	0.592^**^	0.459^**^	0.674^**^	1	0.706^**^
Total score	0.687^**^	0.679^**^	0.653^**^	0.743^**^	0.741^**^	0.726^**^	0.595^**^	0.752^**^	0.678^**^	0.653^**^	0.534^**^	0.572^**^	0.615^**^	0.672^**^	0.637^**^	0.565^**^	0.574^**^	0.718^**^	0.785^**^	0.704^**^	0.703^**^	0.706^**^	1

**Content validity index (CVI)**.

As shown in Supplementary Tables 4, the CVI of each item in the MPSS was 0.88–1, suggesting that the items were comprehensible to the target users, and the scale’s CVI (S-CVI) was 0.926.

**Reliability analysis**.

The Cronbach’s α coefficient of the total MPSS was 0.940 and that of the three subscales were 0.903, 0.911, and 0.882, respectively. The half-test reliability was 0.825, and the retest reliability was 0.912, indicating good reliability (Supplementary Table 5). Supplementary Table 6 shows the correlation coefficient between the 22 items of the questionnaire and the total score and presents the Cronbach α coefficients after removing an item from the questionnaire, all of which were lower than 0.90 prior to removal.

### Validity analysis

#### EFA

KMO was 0.910, and Bartlett’s sphericity test was statistically significant (χ^2^ = 3511.060, *P* < 0.01), indicating that the translated MPSS was suitable for factor structure analysis. A total of three factors with eigenvalues > 1 were extracted, and a total of 65.716% of data discrepancies were explained. Furthermore, EFA results showed that each factor load was > 0.4. Therefore, all items were retained. The specific results can be found in Table 3.

**Table 3:** Exploratory factor analysis of the MPSS in Chinese version (n = 200)

Items	factor 1	factor 2	factor 3
A21 Impossibility to complain to someone	0.805		
A8 Adjustment to frequent wake-ups	0.759		
A9 Baby’s irregular patterns of daily sleep	0.754		
A22 Loneliness at home with the baby	0.737		
A18 Lack of time for socializing with friends	0.731		
A10 My fatigue and exhaustion	0.669		
A19 Lack of time for myself	0.650		
A12 Lack of help with the baby and household chores	0.604		
A11The amount of the household chores	0.571		
A5 Baby’s health problems		0.827	
A4 Baby’s development		0.823	
A6 Recognizing the baby’s needs		0.763	
A3 Insufficient milk supply when breastfeeding		0.759	
A2 Baby’s irregular feeding pattern		0.743	
A1 Choosing the appropriate way of feeding the baby		0.625	
A7 Impossibility to soothe a crying or upset baby		0.560	
A15 Insufficient enjoyment in sexual intercourse			0.856
A14 Insufficient frequency of sexual intercourse			0.833
A13 Being uncertain when to resume intercourse after childbirth			0.737
A16 The thought that my partner finds me unattractive			0.713
A20 Physical appearance after childbirth			0.704
A17 The impossibility to return to the pre-pregnancy weight			0.644
Eigenvalues	10.486	2.153	1.595
Cumulative variance contribution rate (%)	23.439	45.415	64.699

#### CFA

**Fig. 2:**
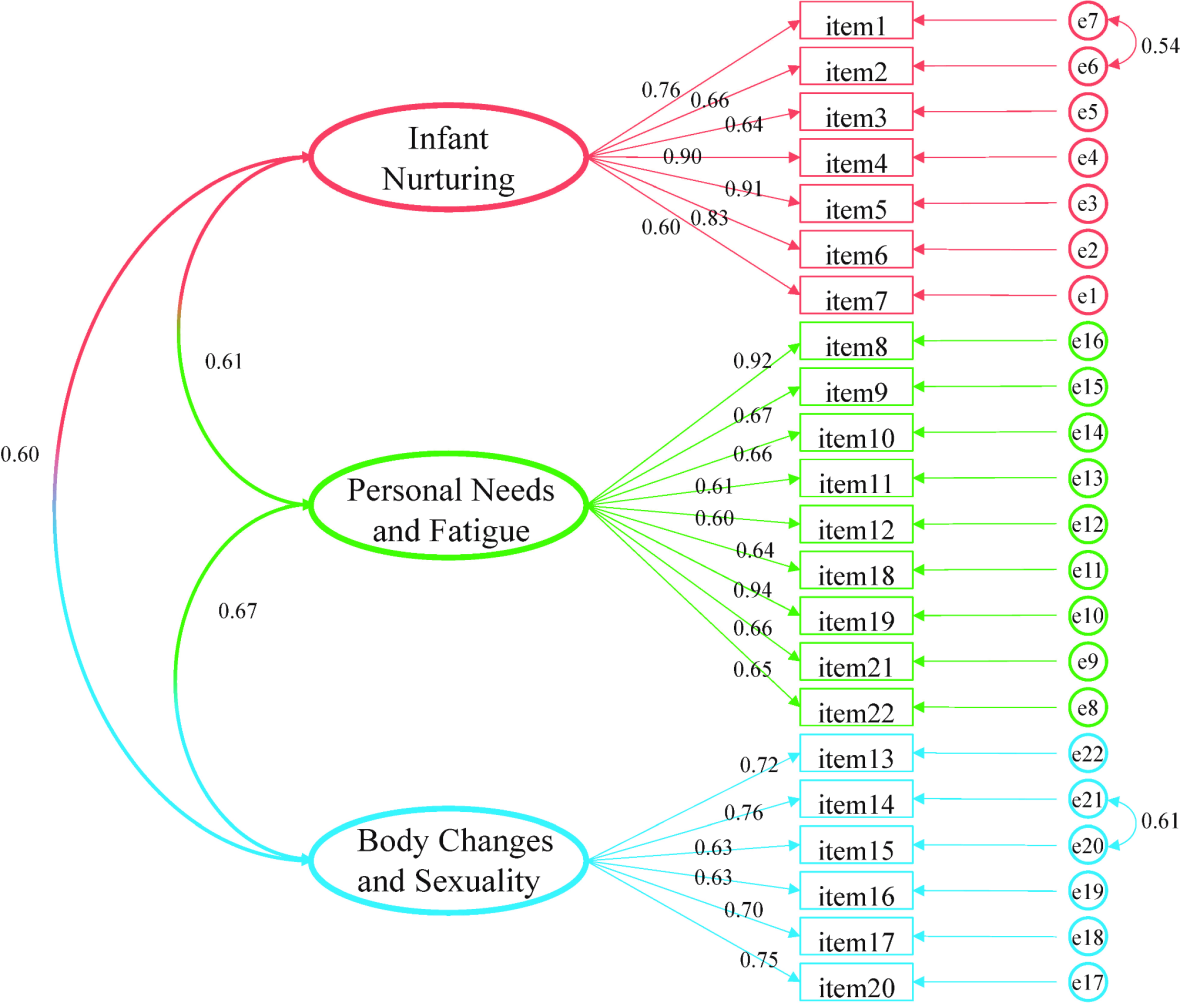
Confirmatory factor analysis model of the Chinese version of MPSS

CFA was used to determine whether the structure of the questionnaire used was consistent with the theoretical structure of the original questionnaire. Furthermore, it was used to validate whether the relationship between each item and the factors is consistent with the hypothesis. The results after model modification are shown in Fig. 2, which indicated that the fittings were good. The values of the indicators were χ2/Df = 2.167(< 3, *P* < 0.05), CFI = 0.918(> 0.9), IFI = 0.919 (> 0.9), TLI = 0.907 (> 0.9), and RMSEA = 0.075 (< 0.08) (Supplementary Table 7).

#### Calibration correlation validity

**Fig. 3:**
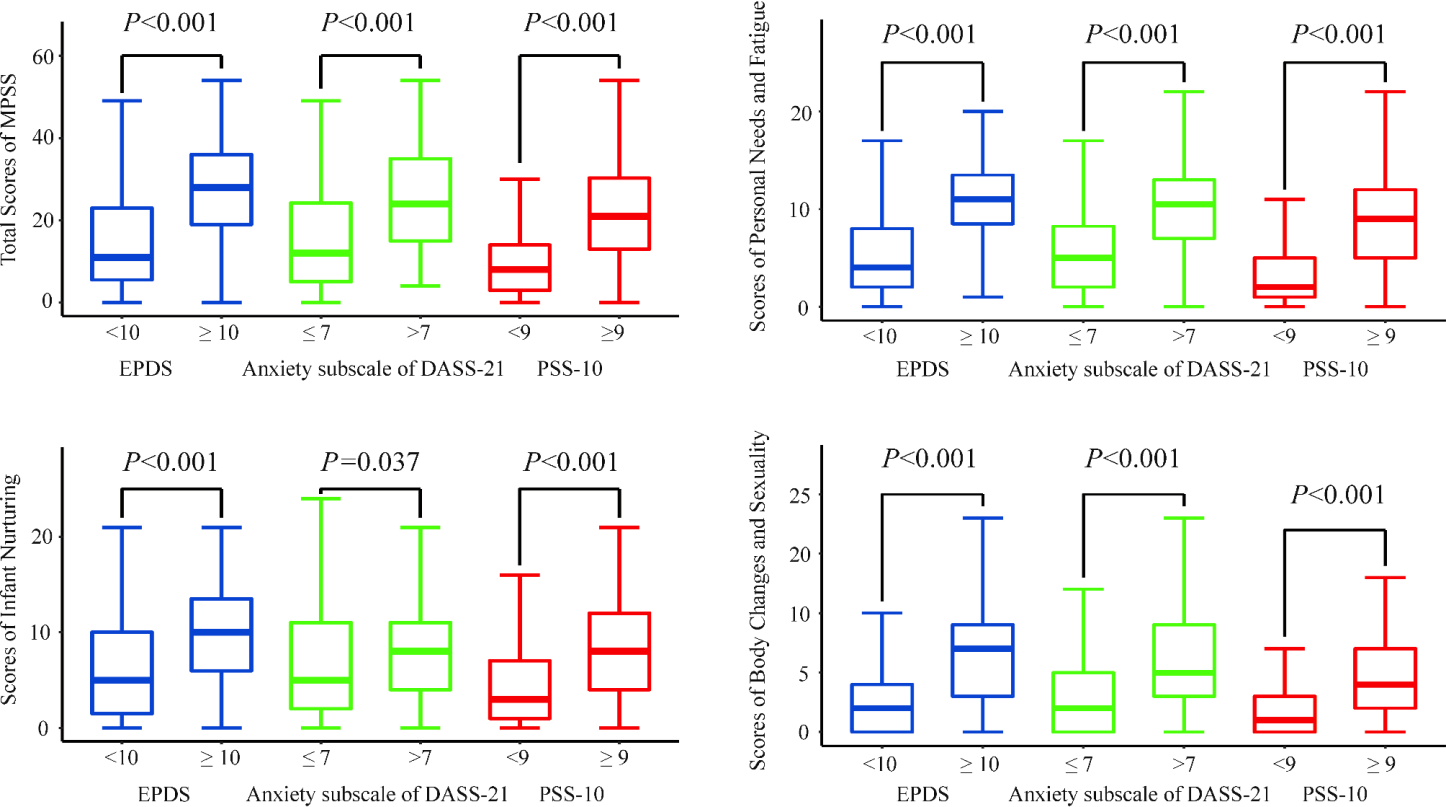
The Scores of MPSS for Maternity in Different Status

**Fig. 4:**
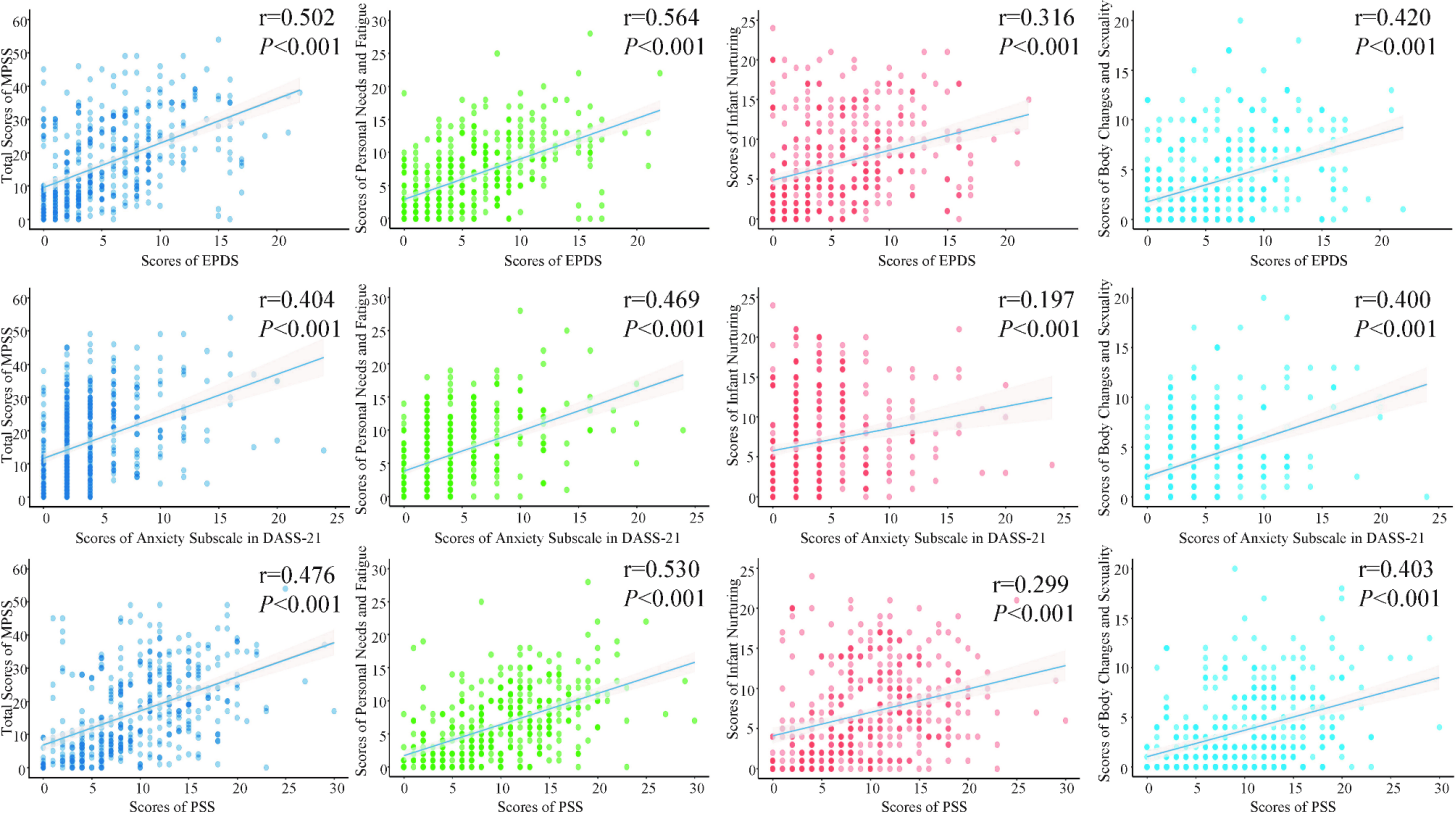
Correlation between EPDS, DASS-21, PSS-10, and the Chinese version of MPSS

EPDS, DASS-21 anxiety dimension, and PSS-10 were used as calibration scales. The EPDS test results (Fig. 3**)** showed that there were 75 women with postpartum depression (18.47%). The MPSS scores of the women with postpartum depression were significantly higher than those with no depression, with a statistically significant difference (*P* < 0.001). Similarly, the MSPP subscale scores of the women with postpartum depression were higher than those with no depression (*P* < 0.001). The results of the anxiety subscale in DASS-21 showed that 68 women (16.75%) were in anxious status. Their MPSS scores were significantly higher compared to those without anxiety (*P* < 0.001), and the MPSS subscales also showed the same trend (*P* < 0.05). The PSS results showed that the MPSS score of the postpartum women with high perceived stress score was significantly higher than those with low perceived stress score (*P* < 0.001), and the MPSS subscales also showed the same trend (*P* < 0.001). The Pearson correlation coefficients between the revised Chinese version of MPSS and EPDS, DASS-21 anxiety dimension, and PSS were 0.502, 0.404, and 0.476 (*P* < 0.001), respectively. The correlation coefficients between each subscale of MSPP and EPDS, DASS-21 anxiety dimension, and PSS are shown in Fig. 4.

### Correlation between the total items of MPSS and each subscale

**Fig. 5:**
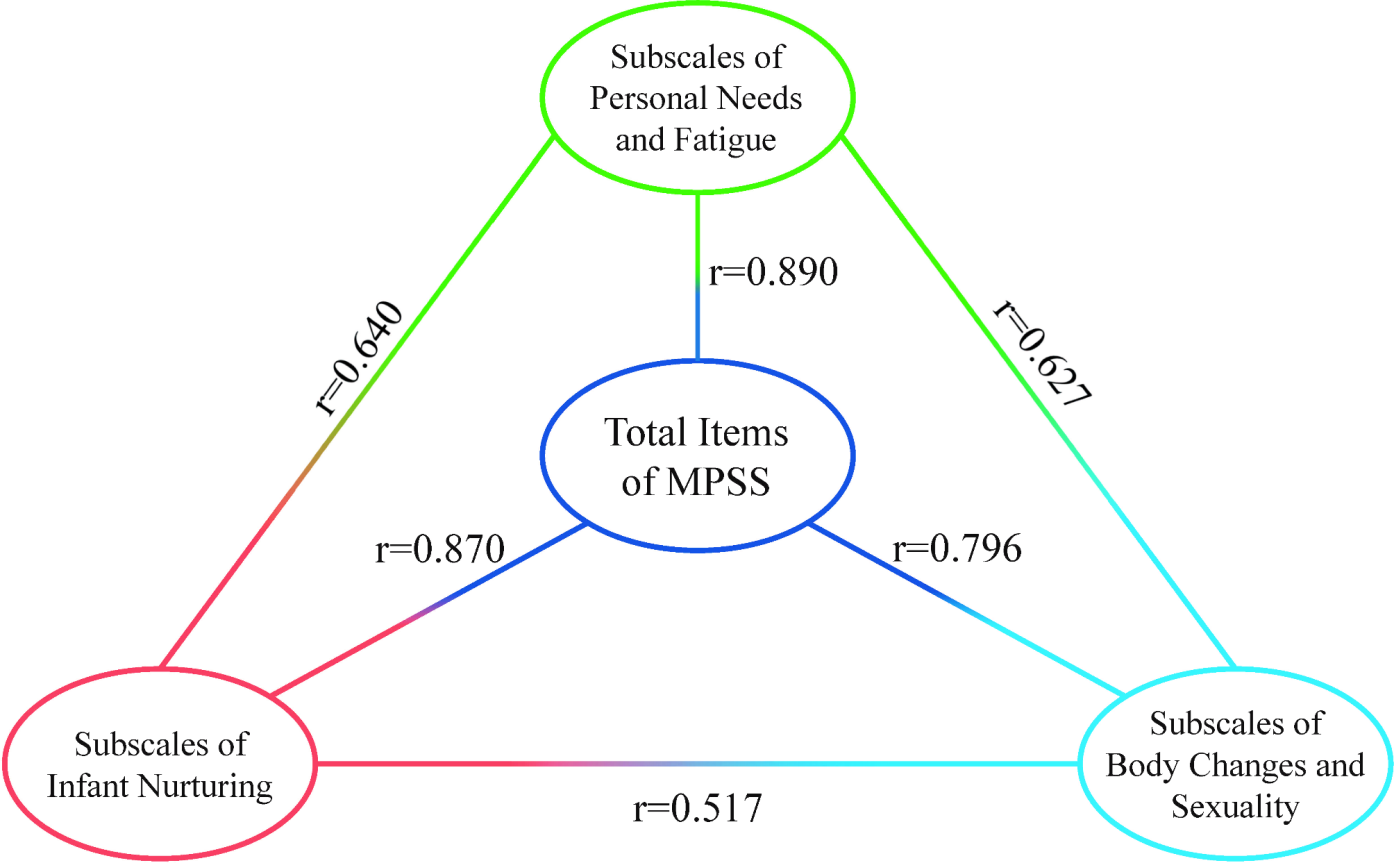
Correlation between total items of MPSS and each subscale

In order of magnitude, each subscale of postpartum stress had an impact on the total score of postpartum stress: personal needs and fatigue (r = 0.890, *P* < 0.001), infant nurturing (r = 0.870, *P* < 0.001), and body changes and sexuality (r = 0.796, *P* < 0.001). Personal needs and fatigue had the closest relationship with infant nurturing (r = 0.640, *P* < 0.001). The relationship between each subscale and the total items MPSS is shown in Fig. 5.

## Discussion

Maternal postpartum stress is an important aspect of postpartum women’s mental health [27]. Although scales that evaluate postpartum stress exist, they lack specificity, and most are universal scales. Even if they have specificity, certain limitations remain, such as the measurement time being too early or too late or too much content [28–30]. At present, there is no specific tool that measures postpartum stress from multiple perspectives. When the MPSS was developed in Croatia, it was only applicable to women within 1 year of childbirth. This is because women in Croatia are entitled to 1-year fully paid maternity leave after childbirth. Therefore, this scale can also be applied to postpartum women in China to measure postpartum stress during maternity leave, thus providing clinical workers with a tool to quickly identify postpartum stress or those at risk. Moreover, this can enable effective and accurate management of postpartum women’s health psychology.

In this study, the guidelines for cross-cultural adaptation of MPSS were strictly followed. We tested the psychometric properties of MPSS in the Chinese population. The MPSS was finally formed through the cultural modification of expert letter consultation and cognitive interview. The 15-member expert panel, which included clinicians and experts in obstetrics and gynecology, nursing, sociology, pediatrics, psychology, and statistics, was highly professional. Given that these members have strong comprehensive strength and comprehensive involvement in the field, it promotes the objectivity and professionalism of the scale evaluation. A formal survey of the included postpartum women showed that the Chinese version of MPSS had good reliability and validity. The translated version of MPSS contains 22 items and 3 subscales. The scale items are moderate, and the completion time is less than 5 min. The contents of the items are clear and easy to understand. Most importantly, the Chinese version of MPSS evaluates postpartum stress from multiple dimensions, including personal needs and fatigue, infant nurturing, and body changes and sexuality. Subsequently, this data can assist medical staff in better evaluating, developing personalized intervention programs, and improving postpartum stress.

The S-CVI of the Chinese version of MPSS was 0.926 > 0.900, and the item CVI was 0.88–1.00 > 0.800. After the cognitive interview, respondents systematically and deeply analyzed the contents of each item of the scale, optimized the scale to adapt it to the Chinese cultural background, and improved the understanding and acceptance of each item of the scale. This indicated that the content validity of the scale was good. In the translated version, saturated with three factors explaining 65.716% of the items’ variance. However, in the original scale, saturated with three factors explaining 36.1% of the items’ variance. In this study, both EFA and CFA suggested that the scale structure was stable. In addition, EPDS and DASS-21 anxiety subscale were applied to evaluate divergent validity, and PSS-10 was applied to evaluate convergent validity. The correlation coefficients between the Chinese version of MPSS and EPDS, DASS-21 anxiety subscale, and PSS-10 were 0.502, 0.404, and 0.476 (*P* < 0.01), respectively. The total scale and all subscales correlated moderately with general perceived stress. Of all, the personal needs and fatigue subscale had the highest correlation with general perceived stress. In line with this finding, some studies suggested that mothers in the postpartum period reported a lack of time for themselves and exhaustion [31]. The total scale and all subscales also correlated with depression and anxiety, in line with the finding of a previous study, which suggested that mothers who experience more stress are at risk for mental health issues [32]. However, it should be noted that the convergent validity was only moderate, with the MPSS and general stress scale sharing only 47.6% of the variance. Similar patterns were seen in other postnatal stresses, suggesting a small overlap between specific and general postnatal stress [33–35].

Reliability reflects the internal consistency and stability of the results measured by the tool. In this study, Cronbach’s α coefficient of the total scale was 0.940, while Cronbach’s α coefficients of the three subscales were 0.903, 0.911, and 0.882, respectively. Cronbach’s α coefficient of the original scale was 0.880, while Cronbach’s α coefficients of the three subscales were 0.850, 0.830, and 0.790, respectively, which were all higher than the original scale. The Cronbach’s α coefficient of all dimensions was greater than 0.8, indicating good internal consistency. The half-point reliability was 0.825 > 0.7, indicating high internal consistency. The retest reliability was 0.912, and the reflection scale has good stability. Overall, the MPSS has good reliability. To our knowledge, this was the first study to validate the tool in different languages. The results of our study may be useful for future research and clinical practice concerning maternal postpartum stress.

As we collected the questionnaire from the postpartum clinic, all the women were the ones who underwent the postpartum check-up 6 weeks after delivery. While also breastfeeding, most postpartum women put all their energy into their children, need more social and family support [36], need intimate relationship support [37], experience increased fatigue, and seldom focus on self-care. These changes influence their identity as mothers. Changes in hormone secretion levels [38] coupled with China’s conservative outlook on sex result in higher stress levels, particularly in personal needs and fatigue and infant nurturing, although body changes and sexuality are higher. Personal needs and fatigue have the closest relationship with infant nurturing, while infant nurturing has the weakest relationship with body changes and sexuality.

Overall, the MPSS demonstrated good internal consistency and significant inter-item and item-total correlations, indicating that the Chinese MPSS could confidently be used to assess maternal postpartum stress. The correlation between MPSS and EPDS, DASS-21 anxiety dimension, and PSS-10 also supports the structural validity of MPSS. The results are consistent with previous research [11]. However, the study has some limitations. First, all the participants were postpartum women from six hospitals in Nantong City, China. Therefore, future studies should include women from other regions to effectively validate the Chinese version of MPSS. Second, because only participants who met certain requirements were selected, there were some deviations in participant selection. Subsequent studies should measure maternal stress at various postpartum time points and compare the maternal stress of postpartum women with multiple births, premature births, advanced age, and different marital statuses. Third, some Pearson’s correlation coefficients in the item analysis section were 0.2–0.4, indicating weak correlation. Further studies with larger sample sizes are warranted to validate this finding. In addition, this is a cross-sectional study, and stress may have existed by chance. Therefore, as emphasized previously, further cohort studies should be conducted to investigate maternal stress at different time points.

## Conclusions

The English version of MPSS was translated into Chinese, and the translated version’s psychometric properties were validated to determine its reliability and validity. The Chinese version of MPSS contains 22 items and 3 subscales, with good reliability and validity. After cultural adaptation, it has been used well among postpartum women in China. The MPSS is expected to provide a scientific basis for the early identification of postpartum women’s stress and formulation of early personalized intervention measures, thus having certain clinical value and practical significance for postpartum women’s physical and mental health.

### Electronic supplementary material

Below is the link to the electronic supplementary material.

Supplementary Material 1

## Data Availability

The raw data of the current study would be available from the corresponding author on reasonable request.

## Abbreviations

MPSS: Maternal Postpartum Stress Scale
EFA: Exploratory factor analysis
CFA: Confirmatory factor analysis
EPDS: Edinburgh Postpartum Depression Scale
DASS-21: Depression, Anxiety, and Stress Scale-21
PSS-10: 10-item Perceived Stress Scale
S-CVI: Content validity index of the scale
HPSS: Hung Postpartum Stress Scale
I-CVI: Item Content Validity Index
IFI: Increment fitting index
TLI: Tucker Lewis index
CFI: Comparative fitting index
